# Commentary on Eskreis-Winkler and Fishbach (2019): A Tendency to Answer Consistently Can Generate Apparent Failures to Learn From Failure

**DOI:** 10.1177/09567976251333666

**Published:** 2025-04-21

**Authors:** Stephen A. Spiller

**Affiliations:** UCLA Anderson School of Management

**Keywords:** replication, model, learning, methodology, confound

## Abstract

Recent research suggests that failure undermines learning: People learn less from failure (vs. success) because failure is ego-threatening and causes people to tune out. I argue that the core paradigm (the Script Task) provides a confounded test of that claim. When people do not learn from test feedback, they may give internally consistent answers on a subsequent test. The Script Task’s scoring guidelines mark consistent answers as correct following success but incorrect following failure. As a result, differences in performance between conditions may result from equivalent learning combined with consistent responding when people do not learn. A descriptive mathematical model shows that lower performance alone is insufficient to conclude that people learn less. An experiment with U.S. Amazon Mechanical Turk workers demonstrates that a retroactive manipulation without feedback replicates the effect. Because the effect of failure on performance is confounded with consistency, the Script Task is not diagnostic regarding whether people learn less from failure unless consistency is ruled out.

Recent research has suggested that people learn less from failure than from success because the threat from failure causes them to tune out (Eskreis-Winkler & Fishbach, 2019). Subsequent research has replicated the effect using the core paradigm (the Script Task; Eskreis-Winkler et al., 2024; Gok & Fyfe, 2024; Keith et al., 2022). I show that the test of the effect of failure is perfectly confounded with the test of participants’ tendency to respond consistently.

**Statement of Relevance**

Prior research has proposed that people learn less from failure than from success because the threat from failure feedback leads them to tune out. This provocative finding is of general interest, not only to researchers across subfields of psychology, but also to the general public. It has been well cited, has been discussed in practitioner-oriented publications, and provides a paradigm (the Script Task) that multiple independent research teams use to understand failures to learn from failure. In the Script Task, learning is operationalized as test performance. But the scoring guidelines used to assess performance in the Script Task mean that a plausible alternative explanation is equally compatible with the data. The effect of failure on performance is confounded with people’s tendency to give consistent responses across multiple tests. Uniform learning coupled with a uniform tendency to respond consistently when people do not learn can generate an apparent failure to learn from failure.

## The Script Task

The Script Task from Eskreis-Winkler and Fishbach’s (2019) Study 2a exemplifies the core paradigm; see Table 1. Participants were randomly assigned to the success condition or the failure condition. An initial Round 1 quiz provided an opportunity to learn through feedback. Participants answered the question, “Which of the following characters in an ancient script represents an animal?” by selecting ᛚ or ᛖ. Regardless of their answer, success participants were told, “You answered this question correct!” and failure participants were told, “You answered this question incorrect!” Participants then answered two more questions, regarding a person (ᛇ, ᚾ) and a bird (ᛗ, ᛉ), and received the same condition-specific feedback after each.

**Table 1.** Script Task With Example Correct Answers, Consistent Responses, and Scores by Condition

Round	Item	Success condition	Failure condition
Round 1	Question 1	Which of the following characters in an ancient script represents an animal? ᛚ or ᛖ	
	Potential guess	ᛚ	ᛚ
	Feedback	Correct	Incorrect
	Implied correct answer	Animal = ᛚ	Animal = ᛖ
	Question 2	Which of the following characters in an ancient script represents a person? ᛇ or ᚾ	
	Potential guess	ᚾ	ᚾ
	Feedback	Correct	Incorrect
	Implied correct answer	Person = ᚾ	Person = ᛇ
	Question 3	Which of the following characters in an ancient script represents a bird? ᛗ or ᛉ	
	Potential guess	ᛉ	ᛉ
	Feedback	Correct	Incorrect
	Implied correct answer	Bird = ᛉ	Bird = ᛗ
Round 2	Question 1	Which of the following characters represents a non-living, stationary object? ᛚ or ᛖ	
	Implied correct answer	Animal = ᛚ, so object = ᛖ	Animal = ᛖ, so object = ᛚ
	Response if learned symbol for animal	ᛖ	ᛚ
	Response if guessed consistently	ᛖ	ᛖ
	Question 2	Which of the following characters represents a non-living, stationary object? ᛇ or ᚾ	
	Implied correct answer	Person = ᚾ, so object = ᛇ	Person = ᛇ, so object = ᚾ
	Response if learned symbol for person	ᛇ	ᚾ
	Response if guessed consistently	ᛇ	ᛇ
	Question 3	Which of the following characters represents a non-living, stationary object? ᛗ or ᛉ	
	Implied correct answer	Bird = ᛉ, so object = ᛗ	Bird = ᛗ, so object = ᛉ
	Response if learned symbol for bird	ᛗ	ᛉ
	Response if guessed consistently	ᛗ	ᛗ
Score	If everyone learned each symbol	100%	100%
	If everyone guessed consistently	100%	0%
	If half learned and half guessed consistently	100%	50%

Note: Round 1 guesses depict modal choices in the experiment. Guessing the other option would lead to the same feedback, so both correct answers and consistent responses would be reversed.

In Round 2, participants answered, “Which of the following characters represents a non-living, stationary object?” three times, once for each of the three Round 1 symbol pairs: (ᛚ, ᛖ), (ᛇ, ᚾ), and (ᛗ, ᛉ). The correct Round 2 answers were the complements of the correct Round 1 answers. For success participants, whatever the participant selected (e.g., ᛉ for bird) was deemed correct in Round 1, so the other symbol (i.e., ᛗ) was correct in Round 2. For failure participants, whatever the participant selected (e.g., ᛉ for bird) was deemed incorrect in Round 1, so that same symbol (i.e., ᛉ) was correct in Round 2. Learning was operationalized as Round 2 performance and was approximately 20 percentage points higher after success than failure.

### Differences in performance are confounded with consistency

The Round 2 scorecard depends on Round 1 responses and condition. Regardless of whether participants learn, consistent responses (e.g., ᛉ is a bird, ᛗ is an inanimate object) are deemed correct after success but incorrect after failure. This positively confounds performance with consistency for success participants and negatively confounds performance with consistency for failure participants. The test of failure’s effect on performance is thus perfectly confounded with, and exactly equivalent to, the test of greater-than-chance consistency (Abelson, 1995; Brauer & Judd, 2000; Shaffer, 1977). If people learn equally from success and failure, equal tendencies to respond consistently when they do not learn will generate apparent failures to learn from failure. This is depicted in Table 1.

### Why would people answer consistently?

When participants learn in Round 1, they answer correctly in Round 2. But not everyone learns everything. Performance averaged near 75%. If people guessed randomly when they did not learn, the probability of learning was 50%.^1^ But random guessing is not the only response strategy when someone has not learned. Someone who has not learned (and so cannot truly know the correct answer in this task) may systematically guess instead. Absent learning, why might participants respond consistently? Prior beliefs and measurement present two possibilities.^2^

First, consider belief-induced consistency. Participants may rely on stable, preexisting beliefs to generate answers across rounds. Features that make one symbol a better representation of an animate being (e.g., physical resemblance, sound-shape mapping, or convention) may make it a worse representation of an inanimate object. This can lead to consistent responses.

Second, consider measurement-induced consistency. Taking tests can induce consistency. Beliefs that are initially independent may shift to align with one another through deliberation (e.g., Holyoak & Simon, 1999). Alternatively, people may recruit their Round 1 responses for consideration when answering Round 2 (e.g., Feldman & Lynch, 1988). In either case, responding in Round 1 induces a consistent response in Round 2.

Belief-induced consistency depends on preexisting associations and in principle could be addressed by selecting stimuli for which no individual has any tendency to give complementary answers. Measurement-induced consistency may still arise even with such stimuli. Any type of consistency when people do not learn results in the confound: Consistent responding generates better performance following success than failure.

## Model and Evidence

Consistent responding in the Script Task leads to lower performance following failure. Next, I present a descriptive mathematical model to specify the concern more precisely. Given the arguments above that participants likely respond consistently when they do not learn, I then present an experiment that tested whether participants respond consistently to the Script Task questions when they cannot learn. I retroactively assigned condition after an adapted Script Task with no feedback (and therefore no learning), yet I found an apparent effect on performance. In an extension, I retroactively reassigned condition labels in the original studies’ datasets and replicated the same apparent effect.

## A Descriptive Mathematical Model of Performance in the Script Task

### Model

After each Round 1 answer, participants receive feedback. On the basis of that feedback, there is some probability that they learn the meaning of the symbol matching the Round 1 concept.^3^ Call the probability of learning from feedback, averaged across success and failure, λ . Call the additional probability of learning from success, beyond learning on average, δ . The probability of learning from success is then λ+δ and the probability of learning from failure is λ−δ . If people learn equally from either, δ=0 and the probability of learning after any feedback is λ .

If participants learn the implied meaning of the symbol matching the Round 1 concept, then they answer the corresponding Round 2 question correctly.^4^ Even if they do not learn the implied meaning of the target symbol, people may still answer the corresponding Round 2 question systematically (e.g., by guessing systematically rather than randomly). Call the probability of giving an internally consistent answer in Round 2 conditional on not learning the implied meaning of the target symbol ρ .

Recall that consistent answers are scored as correct following success but incorrect following failure. The probability that people answer correctly in Round 2 after success, P(correct|success), is (probability learned) + (probability did not learn) × (probability respond consistently conditional on having not learned) = (λ+δ)+(1−(λ+δ))ρ . The probability that people answer correctly in Round 2 after failure, P(correct|failure), is (probability learned) + (probability did not learn) × (probability do not respond consistently conditional on having not learned) = (λ−δ)+(1−(λ−δ))(1−ρ) .

### Results

The difference between performance in the success condition and performance in the failure condition is then given by (2ρ−1)(1−λ)+δ . If δ=0 and people learn equally well from success or failure, the difference is (2ρ−1)(1−λ) . There are four key related results.

#### Any result can be represented by multiple parameter configurations

With three parameters determining performance and only two conditions, the parameters are not uniquely identified: any pattern of results has multiple interpretations. For example, success performance of 85% and failure performance of 65% is consistent with the original explanation: greater learning from success than failure ( λ=0.5 , δ=0.2 ) and no systematic consistency ( ρ=0.5 ). But it is also consistent with equal learning from success or failure ( λ=0.5 , δ=0 ) and high consistency ( ρ=0.7 ).^5^

#### Reduced performance does not imply reduced learning

Following from this first result, observing that performance after failure is lower than performance after success is not sufficient to conclude that there is less learning after failure than there is after success (i.e., that δ>0 ). It enables that conclusion only if one assumes or can prove that there is no consistency conditional on not learning (i.e., that ρ≤0.5 ).

#### Consistent responding can masquerade as a difference in learning

With equal learning from success or failure, performance after failure is lower than performance after success if people answer consistently when they do not learn ( ρ>0.5 ). This could plausibly account for the original effect. For example, for ρ=0.7 , λ=0.5 , and δ=0 , average performance after success is 85% and average performance after failure is 65%.

#### Randomly reassigning condition labels does not change the estimated effect

Recall that the success scorecard is the complement of the failure scorecard. Suppose that before calculating performance, every observation has its condition label flipped: People who received failure feedback are labeled “success” and people who received success feedback are labeled “failure.” As a result, “success” observations (i.e., people who received failure feedback) would be scored according to the success scorecard. Their new scores would be the complement of their true scores: rather than P(correct|failure) , they would be calculated as 1−P(correct|failure) . Similarly, “failure” observations (i.e., people who received success feedback) would be scored according to the failure scorecard. Their new scores would be the complement of their true scores: rather than P(correct|success) , they would be calculated as 1−P(correct|success) .

Perhaps counterintuitively, the difference between scores in the group labeled “success” (which received failure feedback) and scores in the group labeled “failure” (which received success feedback) is again (2ρ−1)(1−λ)+δ . This is the same value, with the same sign, as the difference using correct condition labels.

Given equal cell sizes, any shuffling of condition labels in the raw response data will necessarily result in two subsamples, each balanced between success and failure. In one, observations are scored by the correct scorecard; in the other, observations are scored by the wrong scorecard. For both, the difference in means is (2ρ−1)(1−λ)+δ . The overall difference in means will be a weighted average of those two differences, so the same difference holds for any shuffling of condition labels.^6^ When analyzing results of the Script Task, whether the scorecard used for analysis matches the one implied by the feedback manipulation does not affect the results. Any allocation of condition labels to the raw response data results in the same effect, despite the fact that randomly shuffled condition labels cannot affect learning.^7^

I next test these implications using a version of the Script Task that precludes learning the correct answer.

## Research Transparency Statement

### General Disclosures

**Conflicts of interest:** The author declares no conflicts of interest. **Funding:** This research was supported by funding from the UCLA Anderson School of Management. **Artificial intelligence:** The author used GitHub Copilot and ChatGPT for minor coding tasks. No other artificial-intelligence-assisted technologies were used in this research or the creation of this article. **Ethics:** This research was certified exempt from the relevant Institutional Review Board.

### Experiment disclosures

**Preregistration:** The research question, methods, and primary analysis plan were preregistered (https://researchbox.org/2603) on February 17, 2024, prior to data collection, which began on February 17, 2024. There were minor deviations from the preregistration (for details, see Table S1 in the Supplemental Material available online). **Materials:** All study materials are publicly available (https://researchbox.org/2603). **Data:** All primary data are publicly available (https://researchbox.org/2603). **Analysis scripts:** All analysis scripts are publicly available (https://researchbox.org/2603). **Computational reproducibility:** The computational reproducibility of the results has been independently confirmed by the journal’s STAR Team.

### Posttest disclosures

**Preregistration:** The research question, methods, and primary analysis plan were preregistered (https://researchbox.org/2603) on June 24, 2024, prior to data collection which began on June 24, 2024. There were minor deviations from the preregistration (for details, see Table S1 in the Supplemental Material). **Materials:** All study materials are publicly available (https://researchbox.org/2603). **Data:** All primary data are publicly available (https://researchbox.org/2603). **Analysis scripts:** All analysis scripts are publicly available (https://researchbox.org/2603). **Computational reproducibility:** The computational reproducibility of the results has been independently confirmed by the journal’s STAR Team.

### Experiment S1 disclosures

**Preregistration:** The research question, methods, and primary analysis plan for Experiment S1 were preregistered (https://researchbox.org/2603) on June 24, 2024, prior to data collection, which began on June 24, 2024. There were minor deviations from the preregistration (for details, see Table S1 in the Supplemental Material). **Materials:** All study materials are publicly available (https://researchbox.org/2603). **Data:** All primary data are publicly available (https://researchbox.org/2603). **Analysis scripts:** All analysis scripts are publicly available (https://researchbox.org/2603). **Computational reproducibility:** The computational reproducibility of the results has been independently confirmed by the journal’s STAR Team.

### Experiment S2 disclosures

**Preregistration:** The research question, methods, and primary analysis plan for Experiment S2 were preregistered (https://researchbox.org/2603) on April 5, 2024, prior to data collection, which began on April 5, 2024. There were minor deviations from the preregistration (for details, see Table S1 in the Supplemental Material). **Materials:** All study materials are publicly available (https://researchbox.org/2603). **Data:** All primary data are publicly available (https://researchbox.org/2603). **Analysis scripts:** All analysis scripts are publicly available (https://researchbox.org/2603). **Computational reproducibility:** The computational reproducibility of the results has been independently confirmed by the journal’s STAR Team.

## Experiment

### Method

#### Participants

I aimed to recruit 400 participants from MTurk using CloudResearch’s approved participant pool (Hauser et al., 2023; Litman et al., 2017). This sample size is approximately equal to the largest sample size from the original set of studies (*N* = 402). The dataset included 401 complete observations (225 men, 165 women, 7 nonbinary or third gender, 4 who preferred not to say; after excluding one implausible response, *M_age_* = 43.66 years, *SD_age_* = 13.14). Six participants were missing a response to at least one quiz question, leaving 395 participants for analysis. Attrition and alternate exclusion rules are detailed in the Supplemental Material available online.

#### Design

This experiment was adapted from the original article’s Study 2a (described above and represented in Table 1). There were three changes in addition to the larger sample. First, participants received no feedback, making the participant experience indistinguishable between conditions and precluding participants from learning the correct answer; instructions were adjusted accordingly. Second, condition was assigned retroactively at the end of the experiment, after all measures were collected. Together, these changes made it impossible for condition to affect behavior. Third, answers were not incentivized; instructions were adjusted accordingly. This experiment was certified exempt from the approval of the relevant institutional review board.

### Results

I calculated consistency (proportion of complementary responses) and performance (proportion of correct responses) and regressed each on a contrast-coded variable for retroactive condition label (1 = success, −1 = failure). The key (and the only preregistered) test was the test of condition on performance. The full distribution of consistency (and thus performance), as well as the 2 × 2 contingency table for each question across rounds, is provided in the Supplemental Material.

#### Consistency analysis

Participants’ answers were internally consistent across Rounds 1 and 2, as indicated by the intercept (*M* = 88%, *SD* = 23%; *b* = 0.878, *SE* = 0.012, versus 50%, *t*(393) = 32.65, *p* < .001, Cohen’s *d* = 1.64). As anticipated, given the retroactive random assignment of condition, consistency neither substantively nor significantly varied by condition (success: *M* = 86%, *SD* = 24%; failure: *M* = 89%, *SD* = 22%; *b* = −0.016, *SE* = 0.012; *t*(393) = −1.41, *p* = .158, Cohen’s *d* = 0.14). The null hypothesis of no difference between conditions must be true, as random assignment came after both rounds.

#### Performance analysis

The intercept reveals that average performance did not differ from chance, *M* = 48%, *SD* = 44%, *b* = 0.484, *SE* = 0.012, versus 50%, *t*(393) = −1.41, *p* = .158, Cohen’s *d* = 0.04. Recall that consistency and performance are positively confounded following success but negatively confounded following failure. As a result, because consistency was high in both conditions, performance was substantially and significantly higher in the success condition than in the failure condition (success: *M* = 86%, *SD* = 24%; failure: *M* = 11%, *SD* = 22%; *b* = 0.378, *SE* = 0.012, *t*(393) = 32.65, *p* < .001, Cohen’s *d* = 3.29). Analyses of consistency and performance are precisely equivalent. Because the answer key is flipped across conditions, the test of the intercept against chance for consistency is equivalent to the test of the effect of condition on performance, and the test of the effect of condition on consistency is equivalent to the test of the intercept against chance for performance (Abelson, 1995; Brauer & Judd, 2000; Shaffer, 1977).^8^

As indicated by the model, if condition labels are flipped in the raw response data and scores calculated anew using the scorecards matching the new labels, we reproduce the same signed difference between conditions rather than finding a reversed effect (success: *M* = 89%, *SD* = 22%; failure: *M* = 14%, *SD* = 24%; *b* = 0.378, *SE* = 0.012, *t*(393) = 32.65, *p* < .001, Cohen’s *d* = 3.29). In expectation, any assignment of condition will generate an equivalent raw difference.

#### Extensions

##### Posttest assessing types of consistency

The results above are compatible with belief-induced consistency, measurement-induced consistency, or both. A posttest (*N* = 403) indicated that both may contribute, though possibly differentially across stimuli. The posttest replicated the experiment with a key change: Half of the sample faced the standard order (i.e., animate version of each question in Round 1, inanimate version of each question in Round 2); the other half faced the other order (i.e., inanimate version in Round 1, animate version in Round 2).^9^ Full results are reported in the Supplemental Material.

In each order, more than half of participants gave consistent responses to each version of each question (e.g., ᛉ for bird and ᛗ for inanimate object, or vice versa; *p*s < .001). Supporting a role for belief-induced consistency for question 3, in Round 1 participants tended to select ᛉ for bird and ᛗ for inanimate object (*p*s < .001). There was no such evidence for question 1 or 2. Supporting a role for measurement-induced consistency for questions 2 and 3, the inanimate choice shares elicited in Round 2 differed from those elicited in Round 1 (e.g., the choice share for whether ᛗ or ᛉ represents an inanimate object differed when it followed vs. preceded the question of whether ᛗ or ᛉ represents a bird; *p*s < .001). There was no such evidence for question 1.

Though question 1 answers were internally consistent, neither test of type of consistency was significant. This illustrates the implications of heterogeneity. If half of the population believes ᛚ represents an animal and ᛖ represents an inanimate object and half believes the opposite, the null hypothesis of equal choice shares for each test would be true, despite the presence of belief-induced consistency and the possibility of measurement-induced consistency.

##### The effect of failure for belief-induced inconsistency

Two experiments in the Supplemental Material tested the effect of success versus failure feedback when participants tended to give repeated responses across rounds rather than consistent responses across rounds. Experiment S1 used stimuli selected to induce repeated responding (i.e., systematic inconsistency). As predicted by the model, the effect reversed, revealing an apparent failure to learn from success. Experiment S2 manipulated belief-induced consistency, replicating a failure to learn from failure when the stimuli induced consistency and a failure to learn from success when the stimuli induced inconsistency. The reversal of the effect depending on the stimuli is explainable by consistency, but not by tuning out. Unlike the experiment above, Experiments S1 and S2 enabled learning by providing feedback, demonstrating that consistency still matters when people can learn.

##### Reanalysis of original studies

Using data from each Script Task study from the original article, I reversed condition labels and recalculated performance according to the new scorecard. As the model indicates and the experiment finds, the signed difference in means remains the same (see Table S8 in the Supplemental Material). Relabeling conditions implies using the wrong scorecard. As a result, all correct answers are scored as incorrect and all incorrect answers are scored as correct, thereby reversing the difference. Because the difference between groups is reversed again because of relabeling, the original difference (now twice reversed) reappears. Shuffling labels is similarly ineffectual (see Table S9 in the Supplemental Material). If a researcher had access to raw question responses but not condition labels, any retroactive assignment of condition labels would generate the same apparent effect, because the difference in performance is confounded with the level of consistency.

The Supplemental Material details how a related set of concerns can account for each of the results reported in the original article.

## Discussion

Any tendency toward consistency will induce an apparent effect of failure on performance in the Script Task. Prior theory suggests that people are likely to respond consistently. The experiment indicates that when they receive no feedback and cannot learn, participants do respond consistently. Whereas the confound with consistency is a mathematical necessity, the extent to which consistency holds may vary across different populations. The scoring guidelines mean that reversing or shuffling condition labels reproduces the original effect. Together, these results offer a plausible alternative explanation for apparent failures to learn from failure. The fact that failure reduces performance in the Script Task does not mean that failure reduces learning. Determining failure’s effect on learning requires making strong assumptions, ruling out any role of consistency, or using a different paradigm.

## Supplemental Material

sj-pdf-1-pss-10.1177_09567976251333666 – Supplemental material for Commentary on Eskreis-Winkler and Fishbach (2019): A Tendency to Answer Consistently Can Generate Apparent Failures to Learn From Failure Supplemental material, sj-pdf-1-pss-10.1177_09567976251333666 for Commentary on Eskreis-Winkler and Fishbach (2019): A Tendency to Answer Consistently Can Generate Apparent Failures to Learn From Failure by Stephen A. Spiller in Psychological Science

## Transparency

*Action Editor:* Clayton Critcher

*Editor:* Simine Vazire

*Author Contributions*

**Stephen A. Spiller:** Conceptualization, Data curation, Formal analysis, Funding acquisition, Investigation, Methodology, Project administration, Resources, Software, Supervision, Validation, Visualization, Writing – original draft, Writing – review & editing.

**Artificial Intelligence:** The author used GitHub Copilot and ChatGPT for minor coding tasks. No other artificial-intelligence-assisted technologies were used in this research or the creation of this article.

**Ethics:** This research was certified exempt from the relevant Institutional Review Board.

**Data Availability Statement:** Data, materials, and code are available at https://researchbox.org/2603.

**Open Practices:** General Disclosures. Conflicts of interest: The author declares no conflicts of interest. Funding: This research was supported by funding from the UCLA Anderson School of Management. Artificial intelligence: The author used GitHub Copilot and ChatGPT for minor coding tasks. No other artificial-intelligence-assisted technologies were used in this research or the creation of this article. Ethics: This research was certified exempt from the relevant Institutional Review Board. Experiment disclosures. Preregistration: The research question, methods, and primary analysis plan were preregistered (https://researchbox.org/2603) on February 17, 2024, prior to data collection, which began on February 17, 2024. There were minor deviations from the preregistration (for details, see Table S1 in the Supplemental Material available online). Materials: All study materials are publicly available (https://researchbox.org/2603). Data: All primary data are publicly available (https://researchbox.org/2603). Analysis scripts: All analysis scripts are publicly available (https://researchbox.org/2603). Computational reproducibility: The computational reproducibility of the results has been independently confirmed by the journal’s STAR Team. Posttest disclosures. Preregistration: The research question, methods, and primary analysis plan were preregistered (https://researchbox.org/2603) on June 24, 2024, prior to data collection which began on June 24, 2024. There were minor deviations from the preregistration (for details, see Table S1 in the Supplemental Material). Materials: All study materials are publicly available (https://researchbox.org/2603). Data: All primary data are publicly available (https://researchbox.org/2603). Analysis scripts: All analysis scripts are publicly available (https://researchbox.org/2603). Computational reproducibility: The computational reproducibility of the results has been independently confirmed by the journal’s STAR Team. Experiment S1 disclosures. Preregistration: The research question, methods, and primary analysis plan for Experiment S1 were preregistered (https://researchbox.org/2603) on June 24, 2024, prior to data collection, which began on June 24, 2024. There were minor deviations from the preregistration (for details, see Table S1 in the Supplemental Material). Materials: All study materials are publicly available (https://researchbox.org/2603). Data: All primary data are publicly available (https://researchbox.org/2603). Analysis scripts: All analysis scripts are publicly available (https://researchbox.org/2603). Computational reproducibility: The computational reproducibility of the results has been independently confirmed by the journal’s STAR Team. Experiment S2 disclosures. Preregistration: The research question, methods, and primary analysis plan for Experiment S2 were preregistered (https://researchbox.org/2603) on April 5, 2024, prior to data collection, which began on April 5, 2024. There were minor deviations from the preregistration (for details, see Table S1 in the Supplemental Material). Materials: All study materials are publicly available (https://researchbox.org/2603). Data: All primary data are publicly available (https://researchbox.org/2603). Analysis scripts: All analysis scripts are publicly available (https://researchbox.org/2603). Computational reproducibility: The computational reproducibility of the results has been independently confirmed by the journal’s STAR Team.

## Notes

1. Half of random guesses are correct, and half are incorrect. The 25% of answers that are incorrect represent incorrect guessing, so another 25% represent correct guessing. The remaining 50% represent learning.

2. Other processes can also generate consistency (e.g., alternating responses). Consistent responses generate the confound, no matter the causes.

3. Drawing on the American Psychological Association’s (n.d.) definition, to *learn* in this context means to gain new knowledge from experience. This involves attending to relevant information and integrating it with what is already known. The target to be learned here is the implied meaning of the symbol matching the Round 1 concept (e.g., if one indicates ᛉ for bird, failure feedback implies that ᛗ represents bird). This can be determined by attending to the question, attending to one’s answer, attending to the feedback, and integrating them. Using this knowledge, participants can then answer Round 2 via a process of elimination. Some participants might systematically answer correctly without having learned (e.g., through systematic guessing or preexisting incidentally accurate beliefs), but learning provides a sound basis on which to do so knowledgeably (i.e., the feedback-implied meaning). The model is agnostic regarding the psychological process and consequences of learning, other than the implication that learning will lead to correct answers.

4. This assumption can be relaxed by redefining λ and δ to refer to the joint probability of both learning and using that knowledge, rather than just learning. Both the original article’s proposal and the current model assume that the probability of applying knowledge deduced from feedback is the same across conditions. For the current model, this assumption is merely for simplicity and is not a necessary condition. Differential application of what was learned could provide another possible interpretation of the results (e.g., “I learned that ᛗ means ‘bird’ in Round 1, but everything else I know indicates that ᛉ means ‘bird.’ I trust that broader knowledge base more, so ᛗ must mean ‘inanimate object.’”)

5. Table S2 in the Supplemental Material shows two estimated sets of parameters for each study. One set assumes no systematic consistency in the absence of learning and freely estimates λ and δ . The other set assumes no differential learning from success or failure and freely estimates λ and ρ .

6. In a sample with similar but not equal cell sizes, the difference will be similar but not necessarily equal.

7. Related consequences can occur whenever one differentially transforms data across conditions. Whether such consequences are cause for concern depends on the research question.

8. Apparent differences in Cohen’s *d* are due to use of total standard deviation when calculating the one-sample Cohen’s *d* for levels but pooled standard deviation when calculating the two-sample Cohen’s *d* for differences.

9. I thank the action editor for suggesting this design.

## References

[bibr1-09567976251333666] Abelson R. P. (1995). Statistics as principled argument. Taylor & Francis Group, LLC: Lawrence Erlbaum Associates.

[bibr2-09567976251333666] American Psychological Association. (n.d.). Learning. In APA dictionary of psychology. Retrieved July 6, 2024, from https://dictionary.apa.org/learning

[bibr3-09567976251333666] Brauer M. Judd C. M. (2000). Defining variables in relationship to other variables: When interactions suddenly turn out to be main effects. Journal of Experimental Social Psychology, 36(4), 410–423.

[bibr4-09567976251333666] Eskreis-Winkler L. Fishbach A. (2019). Not learning from failure—the greatest failure of all. Psychological Science, 30(12), 1733–1744. 31702452 10.1177/0956797619881133

[bibr5-09567976251333666] Eskreis-Winkler L. Woolley K. Erensoy E. Kim M. (2024). The exaggerated benefits of failure. Journal of Experimental Psychology: General, 153(7), 1920–1937. 38856918 10.1037/xge0001610

[bibr6-09567976251333666] Feldman J. M. Lynch J. G. (1988). Self-generated validity and other effects of measurement on belief, attitude, intention, and behavior. Journal of Applied Psychology, 73(3), 421–435.

[bibr7-09567976251333666] Gok S. Fyfe E. R. (2024). Learning from failure: The roles of self-focused feedback, task expectations, and subsequent instruction. Journal of Experimental Psychology: General, 153(9), 2328–2344. 39235891 10.1037/xge0001632

[bibr8-09567976251333666] Hauser D. J. Moss A. J. Rosenzweig C. Jaffe S. N. Robinson J. Litman L. (2023). Evaluating CloudResearch’s Approved Group as a solution for problematic data quality on MTurk. Behavior Research Methods, 55(8), 3953–3964. 36326997 10.3758/s13428-022-01999-x PMC10700412

[bibr9-09567976251333666] Holyoak K. J. Simon D. (1999). Bidirectional reasoning in decision making by constraint satisfaction. Journal of Experimental Psychology: General, 128(1), 3–31.

[bibr10-09567976251333666] Keith N. Horvath D. Klamar A. Frese M. (2022). Failure to learn from failure is mitigated by loss-framing and corrective feedback: A replication and test of the boundary conditions of the tune-out effect. Journal of Experimental Psychology: General, 151(8), e19–e25. 10.1037/xge0001170 35951406

[bibr11-09567976251333666] Litman L. Robinson J. Abberbock T. (2017). TurkPrime.com: A versatile crowdsourcing data acquisition platform for the behavioral sciences. Behavior Research Methods, 49(2), 433–442. 27071389 10.3758/s13428-016-0727-z PMC5405057

[bibr12-09567976251333666] Shaffer J. P. (1977). Reorganization of variables in analysis of variance and multidimensional contingency tables. Psychological Bulletin, 84(2), 220–228.

