# Supplementary material for: Commentary on Eskreis-Winkler and Fishbach (2019): A Tendency to Answer Consistently Can Generate Apparent Failures to Learn From Failure
Source: Psychol Sci. 2025 Apr 21;36(11):874–81. doi: 10.1177/09567976251333666 (PMC13428910; doi:10.1177/09567976251333666)
Supplement: sj-pdf-1-pss-10.1177_09567976251333666 – Supplemental material for Commentary on Eskreis-Winkler and Fishbach (2019): A Tendency to Answer Consistently Can Generate Apparent Failures to Learn From Failure [file sj-pdf-1-pss-10.1177_09567976251333666.pdf]

Supplemental Materials for "Commentary on Eskreis-Winkler and Fishbach (2019): A Tendency to Answer Consistently Can Generate Apparent Failures to Learn From Failure"

This supplement provides a list of minor deviations from the preregistrations (see Table S1), as well as additional details regarding the model, the experiment, and the posttest described in the main text. It then presents two additional experiments, an accompanying pretest, and reanalysis of data from the original paper's studies.

Table S1

Minor Deviations from Preregistrations

#	Experiment	Deviation Description	Judgment of Impact
1	Experiment	The preregistration did not address handling of missing quiz responses among observations assigned to a condition or address responses with a duplicate identifier.	Minimal or none. Few observations are impacted. The supplement describes alternative approaches and notes that they all lead to the same conclusions.
2	Experiment	The preregistration did not specify that I would test the intercept in the performance analysis.	Minimal or none. The null hypothesis of no difference from chance is known to be true for this test given retroactive assignment of correct answers. This is included for completeness and to enable reader comprehension of the match to the consistency analysis.
3	Experiment	The preregistration did not specify that I would analyze consistency.	Minimal or none. Due to a mathematical identity, this is precisely equivalent to the performance analysis.
4	Experiment	The preregistration did not specify that I would analyze the data switching the labels.	Minimal or none. Due to a mathematical identity, this is precisely equivalent to the performance analysis.
5	Posttest	The preregistration only imprecisely specified the replication.	Minimal or none. The posttest was designed to examine types of consistency, but it also affords an opportunity to replicate the main experiment. The preregistration did not detail the analysis, so the

			constraints are imposed by the main experiment analysis instead.
6	Posttest	The preregistration did not specify that I would analyze question-level consistency as a function of condition.	Minimal or none. I report for completeness, but it has little to no impact on the main conclusions of the paper.
7	Experiment S1	The preregistration did not address handling of observations with duplicate identifiers.	Minimal or none. Few observations are impacted. The supplement notes that including or excluding them lead to the same conclusions.
8	Experiment S1	The preregistration did not specify that I would examine Round 1 responses as a function of condition (reported under “strategic guessing”).	Minimal. This is an exploratory analysis and does not impact the main argument of the paper in any way.
9	Experiment S2	The preregistration did not address handling of duplicate identifiers.	Minimal. Few observations are impacted. The supplement notes that including or excluding them lead to the same substantive conclusions. The only substantive differences are regarding the exploratory strategic guessing analyses in which p values for some results cross .05 or .10 thresholds.
10	Experiment S2	The preregistration did not specify that I would analyze consistency.	Minimal or none. Due to a mathematical identity, this is precisely equivalent to the performance analysis.
11	Experiment S2	The preregistration did not specify that I would test the intercept, the main effects, or the simple effects in the performance analysis.	Minimal. The interaction provided the key test. The simple effects replicate the original effect or the reversal in Experiment S1. The main effects require interpretation in light of the interaction and simple effects.
12	Experiment S2	The preregistration did not specify that I would examine Round 1 responses as a function of condition (reported under “strategic guessing”).	Minimal. This is an exploratory analysis and does not impact the main argument of the paper in any way. The results in this study are weaker than in S1, and their replicability is uncertain.

A Descriptive Mathematical Model of Performance in the Script Task

Table S2 presents the parameter values from the model that would generate condition means for each study from Eskreis-Winkler and Fishbach (2019) and from the commentary

under two different assumptions. The first assumption is the alternative explanation proposed here, in which $\delta = 0$ (i.e., no differential learning) and ρ (consistency, conditional on not learning) and λ (average learning) are freely estimated. The second assumption is the original paper's proposed psychological process, in which $\rho = 0.5$ (i.e., no systematic consistency) and λ and δ are freely estimated. Both assumptions lead to the same estimate of λ. Either set can perfectly capture the performance results because the three-parameter model is under-identified given two group means. It is also possible that δ takes some value other than 0 and ρ also takes on some value other than 0.5. That is, the values in the table are not the only possibilities.

Several properties of these parameter estimates are worth remarking on:

1. Average learning in commentary Experiment S2 using the original Round 1 stimuli with feedback ($\lambda = 0.50$) is similar to average learning in the original studies ($\lambda = 0.51$).
2. Learning varies across studies in a sensible way. λ is typically around 0.5. It drops to around 0 when there is no feedback (-0.01 across the commentary experiment and posttest; the negative probability can be attributed to sampling error). It increases to 0.67 when incentives are 15 times larger (increased from \$0.10 per correct answer to \$1.50 per correct answer in original Study 2b). There is no apparent difference in λ when no incentives are offered in the core task (0.49, averaged across original Study 1S, commentary Experiment S2) or when baseline incentives (\$0.10) are offered in the core task (0.50, averaged across original Study 2a, 2a replication, 4, 5 self condition, 3S self condition, 4S next condition). Learning is positive but lower in commentary Experiment S1 (0.27), perhaps reflecting the selection of stimuli for which there are very strong, and perhaps stubborn, prior beliefs.

Table S2

Model Parameters Implied by Condition Means Under Two Different Assumptions

Study	Failure *M*	Success *M*	λ	$\rho\|\delta = 0$	$\delta\|\rho = 0.5$	Task description
EWF1[a]	0.48	0.62	0.10	0.58	0.14	Ground truth
EWF2a	0.59	0.8	0.39	0.67	0.21	Core task
EWF2aRep	0.66	0.88	0.54	0.74	0.22	Core task
EWF2b	0.77	0.9	0.67	0.70	0.13	Higher incentives
EWF2c	0.51	0.81	0.32	0.72	0.30	Which is wrong
EWF2d	0.67	0.91	0.58	0.79	0.24	Engagement task
EWF3[a]						Recall task
EWF4	0.68	0.88	0.56	0.73	0.20	Core task + mediator
EWF5self	0.69	0.83	0.52	0.65	0.14	Core task within
EWF5other[a]	0.8	0.82	0.62	0.53	0.02	Eliminates problem
EWF1S	0.63	0.85	0.48	0.71	0.22	Core task
EWF2S[a]						Recall task
EWF3Sself	0.58	0.8	0.38	0.68	0.22	Core task within
EWF3Soth[a]	0.64	0.68	0.32	0.53	0.04	Eliminates problem
EWF4Snext	0.7	0.93	0.63	0.81	0.23	Core task
EWF4Ssame	0.67	0.83	0.50	0.66	0.16	Same page feedback
Average	0.65	0.86	0.51	0.71	0.21	W/out 1,3,5o,2S,3So
Comm1	0.11	0.86	-0.03	0.86	0.75	Placebo
Comm1PAI	0.09	0.88	-0.03	0.88	0.79	Posttest Animate 1st
Comm1PIA	0.24	0.80	0.04	0.79	0.56	Posttest Inanimate 1st
CommS1	0.72	0.55	0.27	0.38	-0.17	Inconsistent stimuli
CommS2Hi	0.54	0.93	0.47	0.87	0.39	High consistency
CommS2Lo	0.84	0.69	0.53	0.34	-0.15	Low consistency

Note. λ is the same under either assumption. EWF refers to studies from Eskreis-Winkler & Fisbach (2019). Comm refers to experiments from this commentary.
[a] Rows with grayed text excluded from Average due to relevant task differences.

3. The average value of $\rho|\delta = 0$ in the original studies (0.71) is compatible with the values in the baseline and high-consistency commentary conditions (0.86 in the experiment; 0.88 and 0.79 in the posttest; 0.87 in the Experiment S2 high-consistency condition).
4. The estimated values of $\rho|\delta = 0$ in the commentary low-consistency conditions (0.38 in Experiment S1, 0.34 in Experiment S2) are consistent with low consistency.
5. The values of $\delta|\rho = 0.5$ in the commentary experiment and posttest (0.75, 0.79 and 0.56) would lead to impossible negative probabilities of learning after failure. They are also

difficult to reconcile with the retroactive placebo manipulation, as there was no difference in the participant experience between conditions. In contrast, the corresponding values of $\rho|\delta = 0$ (0.86, 0.88, and 0.79) are easy to reconcile with the retroactive placebo manipulation.

6. The values of $\delta|\rho = 0.5$ in commentary Experiments S1 (-0.17) and S2 low-consistency (-0.15) are difficult to reconcile with the original psychological process. In contrast, the values of $\rho|\delta = 0$ (0.38 and 0.34) are easy to reconcile with the proposed model.

Experiment: Additional Details

In addition to 401 complete observations, there were another 40 abandoned surveys. They all abandoned the study prior to condition assignment, which occurred at the end of the study.

The main results exclude any observation with an Amazon worker identifier that appeared multiple times in the 401 complete observations. None did.

Alternatively, one may use a more-restrictive approach and exclude any observation with an Amazon worker identifier or an IP address that is repeated in any of the 441 partial or complete observations in addition to excluding responses with missing data. This leads to 389 analyzable observations. No results change.

Alternatively, one can use the original rule, but also calculate consistency and performance using the average across the non-missing responses to include all 401 observations that completed the study, including cases with partially missing data. No results change.

In the preregistration I specified that I would provide the full distributions of consistency. These are provided in Table S3.

Table S3

Experiment Results

	Consistent		Inconsistent		*M* % Consistent	*M* % Correct
	3/3	2/3	1/3	0/3		
Success	138	41	13	5	86%	86%
Failure	152	32	11	3	89%	11%
Quiz 1 Question 1						
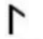	187		39		83%	
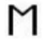	139		30		82%	
Quiz 1 Question 2						
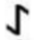	117		16		88%	
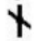	233		29		89%	
Quiz 1 Question 3						
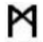	37		19		66%	
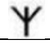	327		12		96%	

Posttest

Method

Participants

I aimed to recruit 400 participants from Amazon Mechanical Turk using CloudResearch's approved participant pool (Hauser et al. 2023; Litman et al. 2017). As with each experiment, this sample size is approximately equal to (or, for Experiment S1, exceeds) the largest sample size from the original set of studies ($N = 402$). The dataset included 403 complete observations (191 men, 201 women, 4 non-binary or third gender, 7 preferred not to say; $M_{age} = 43.67$, $SD_{age} = 13.06$). An additional 32 observations abandoned the survey prior to being assigned to a placebo condition. Of these, 28 abandoned prior to being assigned to an order condition, and only 4 abandoned after being assigned to an order condition.

Design

This posttest was the same as the experiment in the main text with two variations. First, half of participants completed the animate questions in Round 1 and inanimate questions in

Round 2 (as in the experiment and the original paper) whereas the other half completed the inanimate questions in Round 1 and animate questions in Round 2. Second, the order of questions within each round was randomized for each participant. This experiment was certified exempt from the relevant IRB.

Results

Replicating Key Results

Responses exhibited substantively and statistically significant levels of consistency across rounds (M = 84%, SD = 26%; vs. 50%: $t(402) = 26.39$, $p < .001$). Consistency was greater in the animate first condition (M = 90%, SD = 19%) than in the inanimate first condition (M = 78%, SD = 30%; $t(401) = 4.77$, $p < .001$).

As a result, performance in the success placebo group (M = 84%, SD = 25%) exceeded performance in the failure placebo group (M = 17%, SD = 26%; $t(401) = 26.36$, $p < .001$). This difference was greater in the animate first condition ($M_{Success}$ = 88%, $SD_{Success}$ = 20% vs. $M_{Failure}$ = 9%, $SD_{Failure}$ = 18%) than in the inanimate first condition ($M_{Success}$ = 80%, $SD_{Success}$ = 29% vs. $M_{Failure}$ = 24%, $SD_{Failure}$ = 31%; interaction $t(399) = 4.76$, $p < .001$), though both simple effects were statistically significant (ps < .001).

When the consistency and performance analyses are conducted using parallel strategies (as in the experiment), the results are again equivalent (Abelson 1995; Brauer & Judd 2000; Shaffer 1977). The t-test approach above is nearly equivalent, and more-concisely described.

Question-Level Consistency

Each of the three questions regarding symbol pairs exhibited consistency between the animate and inanimate version, whichever came first. See Table S4. In exploratory analyses,

consistency was greater when the animate version came first than when the inanimate version came first.

Table S4

Posttest Question Consistency by Question and Order

	Animate First			Inanimate First			Difference	
	Consistency	$\chi^2(1)$	*p*	Consistency	$\chi^2(1)$	*p*	$\chi^2(1)$	*p*
Question 1	86%	101.74	<.001	79%	66.61	<.001	2.78	.095
Question 2	89%	119.53	<.001	76%	55.62	<.001	9.70	.002
Question 3	95%	159.41	<.001	78%	64.34	<.001	21.38	<.001

Belief-Induced Consistency (Preregistered)

For each version (animate, inanimate) of each question answered in Round 1, I examine whether there was a tendency to favor one response over the other. See Table S5. For question 1 (ᛚ, ᛖ), there was no evidence of a dominant response for either version. For question 2 (ᛇ, ᚽ), both versions led to the same dominant response, suggesting common preexisting beliefs are unlikely to contribute to the effect. For question 3 (ᛗ, ᛘ), each version led to a different dominant response, suggesting belief-induced consistency could contribute to the effect. None of these results can speak to belief-induced consistency coupled with heterogeneous prior beliefs.

Table S5

Posttest Choice Shares by Question Content in Round 1

	Animate in Round 1			Inanimate in Round 1		
	Choice	$\chi^2(1)$	*p*	Choice	$\chi^2(1)$	*p*
Question 1	51% vs. 49%	0.04	.832	54% vs. 46%	1.60	.205
Question 2	35% vs. 65%	18.51	<.001	32% vs. 68%	27.11	<.001
Question 3	11% vs. 89%	119.53	<.001	65% vs. 35%	19.03	<.001

Measurement-Induced Consistency (Preregistered)

For each question, I examine whether the choice proportions vary as a function of whether it was asked in the first round or in the second round (i.e., following the parallel question regarding the complementary concept). Systematic differences would suggest the Round 2 answers were affected by having previously answered Round 1 (i.e., measurement-induced consistency). See Table S6. There is evidence of measurement-induced consistency for questions 2 (ᛇ, ᚽ) and 3 (ᛗ, ᛉ) but not for question 1 (ᛚ, ᛖ). This cannot speak to measurement-induced consistency coupled with heterogeneous prior beliefs.

Table S6

Posttest Changing Choice Share by Whether Question Appears in Round 1 or 2

	Round 1 Choice	Round 2 Choice	$\chi^2(1)$	*p*
Question 1 Inanimate	54% vs. 46%	49% vs. 51%	1.09	.296
Question 2 Inanimate	32% vs. 68%	61% vs. 39%	32.97	<.001
Question 3 Inanimate	65% vs. 35%	89% vs. 11%	30.81	<.001
Question 1 Animate	51% vs. 49%	50% vs. 50%	0.00	.960
Question 2 Animate	35% vs. 65%	57% vs. 43%	19.81	<.001
Question 3 Animate	11% vs. 89%	19% vs. 81%	3.70	.054

Summary

These results suggest there are roles for both belief-induced consistency in the form of common preexisting beliefs (question 3) as well as measurement-induced consistency (questions 2 and 3), with some consistency remaining unexplained (question 1). This suggests each form of consistency may contribute, and the extent to which they contribute varies across stimuli.

There are other differences in Round 2. As they used the original question wordings, the questions were phrased slightly differently (Round 1: "Which of the following characters in an ancient script represents ___?" vs. Round 2: "Which of the following characters represents ___?"), there was a filler question regarding favorite music, and the questions varied in position

in addition to varying in order. While I cannot rule these out as causes, none seem likely contributors to the effect.

Note that the absence of a significant choice-share reversal in Round 1 responses is not indicative of the absence of belief-induced consistency and the absence of a difference between Round 1 and Round 2 choice shares is not indicative of the absence of measurement-induced consistency. Indeed, the high rate of consistency for question 1 must be attributable to some cause. One possibility, which could reconcile either form of consistency with the null effects reported above, is heterogeneous prior beliefs.

Each participant may have strongly-held, consistent prior beliefs even if there is no aggregate tendency to favor one response over the other. For example, if 50% believe ᛚ represents animal and ᛗ represents non-living, stationary object, and the other 50% believe the reverse, belief-induced consistency attributable to heterogeneous prior beliefs could completely explain the effect, despite the two null effects regarding question 1's Round 1 choice shares reported above.

Similarly, the results could be driven by measurement-induced consistency even if there is no difference between Round 1 and Round 2 choice shares. For example, given the same split as in the example above, measurement-induced consistency could lead to answering Round 2 consistently with Round 1, but those choice shares would be the same as the choice shares elicited in Round 1. (Variations on the distribution of prior beliefs and strength of those prior beliefs could account for symmetric or asymmetric consistency between orders.) Here, measurement-induced consistency could completely explain the effect, despite the two null effects regarding comparisons of question 1's first vs. second choice shares reported above.

Note that although the relevance of such heterogeneity is most apparent for question 1, it could readily contribute to consistency for questions 2 and/or 3 as well, in either direction.

Pretest for Experiments S1 and S2

Experiments S1 and S2 vary belief-induced consistency through stimulus-selection. To select appropriate stimuli, I conducted a pretest. I aimed to recruit 100 participants from Amazon Mechanical Turk using CloudResearch's approved participant pool (Hauser et al. 2023, Litman et al. 2017). The dataset included 100 complete observations (47 men, 51 women, 1 non-binary or third gender, 1 preferred not to say; M_{age} = 44.68, SD_{age} = 13.30).

Participants were randomly assigned to complete one of two sets of six questions. Each set of six questions included two questions regarding each pair of symbols (from questions 1, 2, and 3, respectively): one featuring a concept anticipated to favor the first symbol (e.g., knife for ᛚ) and the other featuring a concept anticipated to favor the second symbol (e.g., crown for ᛗ). Each question read "Which of the following characters in an ancient script represents a [knife]? [ᛚ] or [ᛗ]" with response options of "To me, it makes **much more sense** that [ᛚ] represents a [knife];" "To me, it makes **somewhat more sense** that [ᛚ] represents a [knife];" "To me, [ᛚ] and [ᛗ] represent a [knife] **about equally**;" "To me, it makes **somewhat more sense** that [ᛗ] represents a [knife];" and "To me, it makes **much more sense** that [ᛗ] represents a [knife]." The other five concepts in the first set, in addition to *knife* were *crown*, *staircase*, *crossroads*, *net*, and *scarecrow*. In the second set, these were *flag*, *valley*, *cliff*, *knot*, *fence*, and *torch*. Questions were presented one per page in randomized order. At the end, participants reported gender and age. Full distributions are given in Table S7. These results were used to select stimuli for Experiments S1 and S2.

Experiment S1: Reversing the Effect Through Stimulus Selection

The absence of feedback in the experiment could have increased response consistency relative to the original paradigm. Experiment S1 provides feedback as in the original paradigm, but uses concepts selected to generate belief-induced *inconsistency*. Whereas the tune-out effect implies performance will still be lower following failure, belief-induced inconsistency ought to reverse the effect such that performance is higher following failure.

Table S7

Pretest Association of Concepts With Symbols

	Concept	Much more A	Somewhat more A	About equal	Somewhat more B	Much more B
Question 1	Knife	36	9	4	1	0
(ᛚ, ᛖ)	Flag	30	13	3	3	1
	Crown	0	2	1	15	32
	Valley	4	2	7	13	24
Question 2	Staircase	31	15	3	1	0
(ᛇ, ᚾ)	Cliff	23	16	7	3	1
	Crossroads	1	0	3	7	39
	Knot	3	2	4	8	33
Question 3	Net	28	11	4	5	2
(ᛗ, ᛉ)	Fence	35	11	3	1	0
	Scarecrow	0	1	2	12	35
	Torch	1	0	4	11	34

Note. Cells indicate number of participants choosing the column answer for the row concept.

Method

Participants

I aimed to recruit 500 participants from Amazon Mechanical Turk using CloudResearch's approved participant pool (Hauser et al. 2023; Litman et al. 2017). This sample size was slightly larger to account for the expectation that heterogeneity in beliefs could dampen the effect size. The dataset included 498 complete observations (251 men, 243 women, 1 non-binary or third gender, 3 preferred not to say; M_{age} = 42.96, SD_{age} = 13.06). An additional 28 observations

abandoned the study, only 5 of which made it far enough to be assigned to condition (including 2 with duplicate IDs). 4 complete observations had identifiers that were present in the set of incomplete observations. Excluding these observations does not substantively nor significantly change any result.

Design

This study replicated Study 2A from the original paper with two changes. First, there were no incentives; instructions were updated accordingly. As detailed above, the original results with typical incentives were nearly identical to those without any incentives.

Second, while the symbols used as response options were the same as before, the target concepts were changed. Rather than assessing *animal*, *person*, *bird* in Round 1 and *non-living, stationary object* for each question in Round 2, I used stimuli determined by the pretest to favor the same response across rounds for a majority of respondents; see Table S7. Round 1 assessed *knife* (favoring ↾), *knot* (favoring ᚿ), and *torch* (favoring ᛉ) and Round 2 assessed *flag* (favoring ↾), *crossroads* (favoring ᚿ), and *scarecrow* (favoring ᛉ). These stimuli were selected to generate belief-induced inconsistency: in the absence of learning, participants' prior beliefs would lead most of them to give the same response in both rounds. Such a response pattern would lead to correct responses in the failure condition but incorrect responses in the success condition. This experiment was certified exempt from the relevant IRB.

Results

The failure-to-learn effect reversed, such that performance in the failure condition (M = 72%, SD = 34%) exceeded performance in the success condition (M = 55%, SD = 46%; $t(496) = 4.51$, $p < .001$; Cohen's $d = 0.40$). As in other experiments, the consistency and performance analyses provide equivalent results once one accounts for the recoding.

This is consistent with the modeled role of inconsistency and provides an existence proof that the presence of feedback enabling some participants to learn does not negate the potential role of consistency when participants do not learn. Together with Experiment S2 presented next, these results reveal that depending on the stimuli, one may find a positive or negative effect of failure feedback on performance using the Script Task.

Strategic Guessing (Exploratory)

Unexpectedly, Round 1 responses to questions 2 and 3 (but not question 1) exhibited significant differences between feedback conditions. Whereas question 1 responses were equivalent between conditions (success: 96% vs. 4%; failure: 96% vs. 4%; $\chi^2(1) = 0.00$, $p > .9$), question 2 responses (success: 7% vs. 93%; failure: 16% vs. 84%; $\chi^2(1) = 8.99$, $p = .003$) and question 3 responses (success: 5% vs. 95%; failure: 39% vs. 61%; $\chi^2(1) = 83.17$, $p < .001$) significantly differed between conditions. This result is also present in Experiment S2 with the original stimuli, though to a lesser extent.

A plausible explanation is that participants in the failure condition noticed that their initial inclinations were being labeled as incorrect, so they attempted to engage in strategic guessing. Given the study design, that meant chasing a moving target, as the paradigm required that the quiz labeled any choice in the failure condition as incorrect.

Experiment S2: Replicating and Reversing the Effect Through Stimulus Selection

Method

Participants

I aimed to recruit 400 participants from Amazon Mechanical Turk using CloudResearch's approved participant pool (Hauser et al. 2023; Litman et al. 2017). The dataset included 401 complete observations. An additional 47 abandoned the study prior to condition assignment and

12 abandoned the study following condition assignment (Zhou & Fishbach 2016). 14 completed observations included 7 duplicate Amazon identifiers. I exclude these observations, resulting in 387 observations for analysis (174 men, 207 women, 2 non-binary or third gender, 4 preferred not to say; M_{age} = 44.74, SD_{age} = 13.22).

One may instead choose a more-restrictive exclusion, and exclude any observation with an Amazon worker identifier or an IP address that appears multiple times in any of the 460 complete or incomplete observations. This leaves 379 observations for analysis. One may instead choose a less-restrictive exclusion and include all complete observations, including duplicates, leaving 401 observations for analysis. The only minor changes to substantive or statistical inferences are in the exploratory analysis regarding strategic guessing and detailed in footnote 1.

Design

This study was a replication of Study 2a from the original paper, with two changes in addition to the larger sample. First, choices were not incentivized; instructions were adjusted accordingly. As detailed above, the original results with typical incentives were nearly identical to those without any incentives.

Second, the experiment included a manipulation of expected consistency, resulting in a 2 (feedback: success, failure) x 2 (consistency: high, low) between-subjects design. Half of the sample was randomly assigned to complete a Round 2 quiz favoring idiosyncratically-unselected symbols from Round 1, encouraging high consistency. The other half completed a Round 2 quiz favoring idiosyncratically-selected symbols from Round 1, encouraging low consistency. The high-consistency condition should replicate the effect from the original paper, whereas the low-consistency condition should reverse the effect, replicating the effect from Experiment S1. This experiment was certified exempt from the relevant IRB.

Materials

For each of the six Round 1 symbols (two options for each of three concepts) from the original task, a new concept was selected from the pretest that strongly favored that symbol over its complement. For question 1, *knife* favored ᛚ and *crown* favored ᛖ; corresponding concepts were *staircase* for ᛇ and *crossroads* for ᚾ in question 2 and *fence* for ᛞ and *torch* for ᛉ in question 3. Each of these Round 2 concepts can plausibly be considered *a non-living, stationary object* and so is also consistent with the original Round 2 quiz. Participants assigned to the high-consistency condition were tested on concepts that favored their own idiosyncratically-unselected symbols: if a participant responded ᛚ to Round 1 question 1, they would be tested on *crown* in Round 2; if they responded ᛖ to Round 1 question 1, they would be tested on *knife* in Round 2. Participants assigned to the low-consistency condition were tested on concepts that favored their own idiosyncratically-selected symbols: if a participant responded ᛚ to Round 1 question 1, they would be tested on *knife* in Round 2; if they responded ᛖ to Round 1 question 1, they would be tested on *crown* in Round 2. Unlike in other experiments (including Experiment S1), different participants in the same condition received different Round 2 quiz content: the quizzes varied according to both consistency condition and participants' Round 1 answers.

Results

I regressed Round 2 consistency and performance measures on feedback (contrast coded 1 = success, -1 = failure), consistency manipulation (1 = high, -1 = low), and their interaction. The key (and only preregistered) test was the test of the interaction on performance. Estimates of Cohen's *d* for the simple effects in this experiment are calculated using the error only from the corresponding consistency subsample, not using the error from the full sample.

Consistency Analysis

Average consistency, as indicated by the intercept, was 56% (*SD* = 44%), significantly exceeding 50% (b = 0.562, se = 0.017; vs. 50%: $t(383) = 3.69$, $p < .001$). Consistency was higher in the high-consistency condition (*M* = 70%, *SD* = 38%) than the low-consistency condition (*M* = 43%, *SD* = 44%; b = 0.136, se = 0.017, $t(383) = 8.03$, $p < .001$, $\eta_p^2 = 0.14$). Consistency was also higher in the success condition (*M* = 81%, *SD* = 35%) than the failure condition (*M* = 31%, *SD* = 37%; b = 0.251, se = 0.017, $t(383) = 14.88$, $p < .001$, $\eta_p^2 = 0.37$), indicating that people were sensitive to the correct answer. The consistency manipulation did not interact with feedback condition to impact consistency (b = -0.016, se = 0.017, $t(383) = -0.93$, $p = .354$, $\eta_p^2 = 0.002$).

Performance Analysis

Average performance (as given by the intercept) was 75% (SD = 36%), significantly exceeding 50% (b = 0.751, se = 0.017, $t(383) = 14.88$, $p < .001$). There was no statistically significant main effect of the consistency manipulation (low: *M* = 77%, *SD* = 36%; high: *M* = 74%, *SD* = 36%; b = -0.016, se = 0.017, $t(383) = -0.93$, $p = .354$, $\eta_p^2 = 0.002$). There was a significant main effect of feedback such that average performance in the success condition (*M* = 81%, *SD* = 35%) exceeded average performance in the failure condition (*M* = 69%, *SD* = 37%; b = 0.062, se = 0.017, $t(383) = 3.69$, $p < .001$, $\eta_p^2 = 0.03$).

One might choose to interpret this main effect as greater learning from success than from failure. But this effect is equivalent to the tendency toward consistency, so it may instead reflect asymmetric consequences of the consistency manipulation. If, in the absence of learning, the favored symbol in the high-consistency condition is chosen more frequently than is the favored symbol in the low-consistency condition (e.g., due to equal additive effects of measurement-

induced consistency in both conditions), the manipulation will lead to consistency on average, and thus an apparent main effect of feedback on performance.

These main effects were qualified by a significant interaction (b = 0.136, se = 0.017, $t(383) = 8.03$, $p < .001$, $\eta_p^2 = 0.14$). When Round 2 content favored consistent answers, the original finding replicated: participants did better in Round 2 following success (*M* = 93%, *SD* = 21%) than failure (*M* = 54%, *SD* = 38%; b = 0.198, se = 0.024, $t(383) = 8.17$, $p < .001$, Cohen's *d* = 1.30). But when Round 2 content favored inconsistent answers, the effect reversed: participants did better in Round 2 following failure (*M* = 84%, *SD* = 29%) than success (*M* = 69%, *SD* = 41%; b = -0.073, se = 0.024, $t(383) = -3.11$, $p = .002$, Cohen's *d* = 0.41).

As in prior experiments, the consistency and performance results are exactly equivalent when swapping main effects and interactions because of how the answer key is recoded (Abelson 1995; Brauer & Judd 2000; Shaffer 1977). Whether success or failure leads to better performance depends on the content on which learners are assessed.

Strategic Guessing (Exploratory)

Replicating Experiment S1 using the original stimuli, Round 1 responses to questions 2 and 3 (but not question 1) exhibited some indication of differences between feedback conditions. Responses to question 1 did not differ in the success (68% vs. 32%) and failure (65% vs. 35%) conditions ($\chi^2(1) = 0.33$, $p = .566$). But responses to question 2 (success: 41% vs. 59%; failure: 32% vs. 68%; $\chi^2(1) = 3.47$, $p = .062$) and question 3 (success: 18% vs. 82%; failure: 27% vs. 73%; $\chi^2(1) = 3.90$, $p = .048$) showed some indications of differences between conditions.[1] This

[1] The corresponding *p* values for questions 1, 2, and 3 for the more-restrictive sample (*N* = 379) are .663, .057, and .053; for the less-restrictive sample (*N* = 401) they are .574, .105, and .053.

was more pronounced in Experiment S1 than S2, perhaps because there was a stronger and more-uniform tendency to give the dominant response option in Experiment S1 than S2.

Reanalysis of Original Studies with Reversed and Shuffled Condition Labels

The experiment found an apparent effect of retroactive condition assignment without feedback, and that the effect was necessarily the same when reversing condition labels. I next apply the same logic to data from the original paper's Script Task studies with feedback and replicate the same effect on performance when condition labels are reversed or shuffled.

Analysis Strategy

I reanalyzed each of the posted datasets from the original paper that relied on the focal design (Studies 2a, 2a replication, 2b, 2c, 2d, 4, and 5; I address Studies 1 and 3 separately, as their tasks used different structures). Although the posted datasets do not include the question-level responses, they do include condition and performance. From condition and performance, proportion of complementary and repeated responses can be computed for every participant. After reassigning condition labels, I can then compute performance using the method from the original paper. This leads to the same score that one would compute from the question-level data.

Results

As shown in Result 4 of the mathematical model, reversing or shuffling condition labels reproduced the original effect in each dataset.[2] See Tables S8 and S9. To understand the intuition why, consider the experiment presented in the main text. After original performance scores were converted back to consistency scores, average proportion of consistent responses exceeded 50%. Shuffling condition labels does not affect average consistency. Whenever empirical consistency

[2] For perfectly-balanced cells, shuffling condition labels leads to the same raw effect sizes, though standard deviations (and thus test statistics) vary. For nearly-balanced cells (as here), shuffling condition labels leads to nearly the same raw effect sizes, though again standard deviations and thus test statistics vary. The higher standard deviations in Study 2b explain why its effect is the same size but marginally significant in the shuffled analysis.

Table S8

Reanalysis of Original Experiments After Reversing Condition Labels and Rescoring

	"Failure" Label (Success Feedback)	"Success" Label (Failure Feedback)	Comparison[c]
Study 1[a]	62% (26%)	48% (28%)	$t(327) = -4.71, p < .001$
Study 2a	20% (35%)	41% (41%)	$t(97) = 2.80, p = .006$
Study 2a replication	12% (25%)	34% (36%)	$t(323) = 6.17, p < .001$
Study 2b	10% (22%)	23% (31%)	$t(100) = 2.57, p = .012$
Study 2c	19% (38%)	49% (44%)	$t(112) = 3.86, p < .001$
Study 2d	9% (21%)	33% (34%)	$t(101) = 4.30, p < .001$
Study 3[a]	94% (16%)	59% (39%)	$t(98) = -5.90, p < .001$
Study 4	12% (27%)	32% (36%)	$t(298) = 5.65, p < .001$
Study 4 mediator[a]	1.70 (1.15)	3.22 (1.22)	$t(298) = 11.15, p < .001$
Study 5 Self[b]	17% (33%)	31% (38%)	$t(201) = 5.35, p < .001$
Study 5 Other[b]	18% (31%)	20% (32%)	$t(199) = 0.86, p = .393$

Note. Columns report Means (Standard Deviations) given each Label-Feedback condition.
[a] Original analysis of Studies 1, 3, and 4 mediator did not involve recoding.
[b] Success vs. failure was manipulated within-subject in Study 5.
[c] t tests are signed such that positive values represent a higher mean in the "Success" Label group than "Failure" Label group to align with the original paper's Table 1.

Table S9

Reanalysis of Original Experiments After Shuffling Condition Labels and Rescoring

	"Failure" Label, Randomly Shuffled	"Success" Label, Randomly Shuffled	Comparison[c]
Study 1[a]	57% (28%)	53% (28%)	$t(327) = -1.31, p = .192$
Study 2a	35% (44%)	57% (42%)	$t(97) = 2.57, p = .012$
Study 2a replication	37% (40%)	59% (41%)	$t(323) = 4.92, p < .001$
Study 2b	45% (43%)	60% (43%)	$t(100) = 1.75, p = .084$
Study 2c	29% (43%)	60% (45%)	$t(112) = 3.80, p < .001$
Study 2d	33% (38%)	56% (42%)	$t(101) = 2.96, p = .004$
Study 3[a]	71% (37%)	82% (32%)	$t(98) = 1.54, p = .126$
Study 4	42% (43%)	63% (41%)	$t(298) = 4.45, p < .001$
Study 4 mediator[a]	2.45 (1.40)	2.44 (1.42)	$t(298) = -0.03, p = .978$
Study 5 Self[b]	43% (45%)	57% (43%)	$t(201) = 5.35, p < .001$
Study 5 Other[b]	49% (44%)	52% (44%)	$t(199) = 0.86, p = .393$

Note. Columns report Means (Standard Deviations) given each Label condition.
[a] Original analysis of Studies 1, 3, and 4 mediator did not involve recoding.
[b] Success vs. failure was manipulated within-subject in Study 5.
[c] t tests are signed such that positive values represent a higher mean in the "Success" Label group than "Failure" Label group to align with the original paper's Table 1.

exceeds 50%, performance for success will exceed performance for failure because of the use of different scorecards (whether or not those scorecards are the proper scorecards). This analysis thus mimics the main Experiment. Based on these results, one might wrongly infer that randomly shuffled-condition labels affected learning.

Two aspects of these results deserve further comment. First, whereas the difference is the same, aggregate performance levels are considerably lower. This is simply a result of the usual effect of reversing labels, in which the average is the same and the difference is reversed. Because the recoding approach converts levels to differences and differences to levels (Abelson 1995; Brauer & Judd 2000; Shaffer 1977), the average is reversed and the difference is the same. Similar logic holds for the shuffled analysis.

Second, these procedures provide different results for studies that do not involve the by-condition recoding (performance in Studies 1 and 3; mediator in Study 4). This is the expected result for a typical test. There was also no difference for the comparison of success vs. failure within Study 5's Other condition. This simply reproduces the null effect from the original study.

These results depend on the empirically observed consistency, whether it is derived from failure to learn from failure or consistent responding in the absence of learning. But because the results are the same whether one uses true condition assignment, or reversed condition labels, or randomly-shuffled condition labels, the effect is independent of the feedback manipulation.

Concerns Regarding Each Study From the Original Paper

The argument in the main text directly addresses Eskreis-Winkler and Fishbach's (2019) Script Task as it applies to Studies 2a, 2a replication, 2b (with larger incentives), and 2d (the structurally-identical Couples Task). Related concerns can account for the remaining studies.

1. Study 1 used test questions with real answers. Feedback and retesting were selectively targeted to questions that were answered correctly or incorrectly. The same consistency-related concern, particularly belief-induced consistency, applies. Given tailored question selection, relative to participants in the success condition, participants in the failure condition faced questions in the test phase which were idiosyncratically harder and for which their initial inclinations indicated the wrong answer.
2. Study 2c's Round 2 required participants to indicate incorrect (rather than correct) answers. The same consistency-related concern from the main text applies if participants answered by, e.g., determining the correct answer and then selecting the other answer.
3. Study 3 compared failure feedback to no feedback and required participants to recall their prior answers. Participants could have taken two different approaches to responding. First, they could have attempted to recall their prior answers. Second, they could have attempted to reconstruct an answer based on their beliefs, under the reasonable assumption that repeated construction would lead to the same answer. (These approaches could be used together: participants may attempt to recall and, should that fail, resort to reconstruction.) Without feedback, answers recalled from memory of what participants selected and answers reconstructed anew based on their beliefs about the concept-symbol mapping would match and both lead to the correct response. But after failure feedback, if some participants learn something, these approaches will sometimes lead to conflicting answers. Answers recalled from memory of what participants selected would provide the correct response (but the incorrect concept-symbol mapping). Answers reconstructed anew based on updated beliefs following learning would provide the correct concept-symbol mapping (but the incorrect response). If some participants have imperfect recall

and instead attempt to reconstruct the answer anew following partial learning, equal failure to recall and equal partial learning across conditions would lead to more incorrect responses in the failure condition than the no feedback condition.

4. Study 4 tested for statistical mediation by ego-threat. If failure is ego-threatening, and that threat is accentuated for people who respond more-consistently (e.g., for participants who hold stronger pre-feedback beliefs), statistical mediation can result.
5. Study 5 crossed feedback with learning based on feedback for the self vs. someone else. The other condition revealed no feedback effect, which was attributed to eliminating the ego-threat. But independent of any reduction of ego-threat, the other condition also plausibly reduced both belief-induced consistency (as the other's beliefs were independent of participants' own) and measurement-induced consistency (as there was no deliberation to cause beliefs to shift and align), thereby eliminating the confound.

References

Abelson, R. P. (1995). *Statistics as Principled Argument*. Erlbaum.

Brauer, M., & Judd, C. M. (2000). Defining variables in relationship to other variables: When interactions suddenly turn out to be main effects. *Journal of Experimental Social Psychology*, *36*(4), 410-423.

Eskreis-Winkler, L., & Fishbach, A. (2019). Not learning from failure—The greatest failure of all. *Psychological Science*, *30*(12), 1733-1744.

Hauser, D. J., Moss, A. J., Rosenzweig, C., Jaffe, S. N., Robinson, J., & Litman, L. (2023). Evaluating CloudResearch's Approved Group as a solution for problematic data quality on MTurk. *Behavior Research Methods*, *55*(8), 3953-3964.

Litman, L., Robinson, J., & Abberbock, T. (2017). TurkPrime. com: A versatile crowdsourcing data acquisition platform for the behavioral sciences. *Behavior Research Methods*, *49*(2), 433-442.

Shaffer, J. P. (1977). Reorganization of variables in analysis of variance and multidimensional contingency tables. *Psychological Bulletin*, *84*(2), 220-228.

Zhou, H., & Fishbach, A. (2016). The pitfall of experimenting on the web: How unattended selective attrition leads to surprising (yet false) research conclusions. *Journal of Personality and Social Psychology, 111*(4), 493–504.
